# 1745. Respiratory Syncytial Virus Surveillance of Child Care Attendees, Family Members, and Child Care Providers

**DOI:** 10.1093/ofid/ofad500.1576

**Published:** 2023-11-27

**Authors:** Khalil Chedid, Timothy Shope, Andrew Hashikawa, Anna Wang-Erickson, John V Williams, Emily T Martin

**Affiliations:** University of Michigan School of Public Health, 1317 Millbrook Trl, Michigan; University of Pittsburgh School of Medicine, Pittsburgh, Pennsylvania; University of Michigan, Ann Arbor, Michigan; University of Pittsburgh, Pittsburgh, Pennsylvania; University of Pittsburgh, Pittsburgh, Pennsylvania; University of Michigan, Ann Arbor, Michigan

## Abstract

**Background:**

Respiratory syncytial virus (RSV) is a leading cause of severe respiratory illness among young children. In the U.S., a third of these children attend child care centers (CCC), where RSV can spread rapidly and be transmitted to family members. The study objective was to determine the incidence of RSV in CCC attendees and their household contacts.

**Methods:**

The prospective cohort enrolled students (< 6 years old), their household contacts and child care providers (CCP) at six CCC in Ann Arbor, MI from May 2021 to March 2022. Participants completed a symptom diary and weekly nasal swabs. RT-PCR was used to detect RSV. Incidence rates were calculated using Poisson regression.

**Results:**

A total of 2,342 swabs were collected during 19,121 person-days from 32 students, 13 household child contacts (7 of whom were also students), 45 household adult contacts, and 10 CCP. The incidence rates per 10,000 person-days (95% CI) for total vs. asymptomatic cases were 10.2 (4.2, 24.5) vs. 2.0 (0.3, 14.5) for students, 8.4 (2.1, 33.7) vs. 4.2 (0.6, 30.0) for child contacts, and 5.4 (2.2, 12.9) vs. 1.1 (0.2, 7.6) for adult contacts, respectively. Twelve people tested positive for RSV: 5 (15.6%) were students, 2 (15.4%) were child contacts, 5 (11.1%) were adult contacts, and none were CCP. Semiquantitative cycle threshold was comparable between samples from asymptomatic and symptomatic cases. Three students, 1 child contact and 3 adult contacts had serial positive swabs for a single illness. Asymptomatic cases were detected in 1 (20%) student, 1 (20%) adult contact, and 1 (50%) child contact. Suspected transmission events were observed in 3/24 (12.5%) households with documented infection. All cases were detected between 9/6/21 and 1/17/22, coinciding with RSV circulation in Michigan during the study period.

**Conclusion:**

This study demonstrates the continued presence of RSV in CCC, including during times of increased disease mitigation, and transmission between children and household contacts may have occurred. Asymptomatic infection was detected, albeit at lower rates than symptomatic infections. Given the incidence of RSV detected, our findings underscore the importance of implementing additional surveillance and infection prevention policies in CCC to mitigate the impact of RSV on vulnerable populations.

**Disclosures:**

**Khalil Chedid, MPH, MD, PHD**, Merck: Grant/Research Support **Timothy Shope, MD MPH**, Merck: Grant/Research Support **Andrew Hashikawa, MD**, Merck: Grant/Research Support **Anna Wang-Erickson, PhD**, Merck: Grant/Research Support **John V. Williams, MD**, Merck: Grant/Research Support|Quidel: Board Member **Emily T. Martin, PhD, MPH**, Merck: Grant/Research Support

